# Ellagic acid regulates astrocytic responses under inflammatory conditions in primary astrocyte cultures: effects on oxidative stress, inflammatory cytokines, and glycogen synthase kinase-3β

**DOI:** 10.1007/s11033-026-12671-4

**Published:** 2026-09-05

**Authors:** Thais Marini da Rosa, Larissa Menezes da Silveira, Natália Pontes Bona, Juliane Torchelsen Saraiva, Júlia Araújo da Silva, William Borges Domingues, Lucas Petitemberte de Souza, Mariana Cavalcanti Nascimento, Vinicius Farias Campos, Francieli Moro Stefanello, Cinthia Melazzo de Andrade, Nathalia Stark Pedra, Roselia Maria Spanevello

**Affiliations:** 1 Programa de Pós-Graduação em Bioquímica e Bioprospecção, Universidade Federal de Pelotas, Campus Universitário Capão do Leão s/n, Pelotas, RS, CEP 96010-900 Brazil; 2 Programa de Pós-Graduação em Biotecnologia, Universidade Federal de Pelotas, Campus Universitário Capão do Leão, s/n, Pelotas, RS, CEP 96010-900 Brazil; 3 Programa de Pós-Graduação em Medicina Veterinária, Universidade Federal de Santa Maria, Cidade Universitária, Santa Maria, RS CEP 97105-900 Brazil; 4 Programa de Pós-Graduação em Bioquímica Toxicológica, Departamento de Bioquímica e Biologia Molecular, Universidade Federal de Santa Maria, Santa Maria, RS CEP 97105-900 Brazil; 5 Federal University of Santa Maria. Av. Roraima No. 1000, University City, Camobi District, Santa Maria – RS, 97105-900 Brazil

**Keywords:** Ellagic acid, Antioxidant, Interleukin, Astrocytes, Lipopolysaccharide, Neuroinflammation, GSK-3β

## Abstract

**Background:**

Neuroinflammation involves the activation of glial cells, particularly astrocytes, and microglia, in response to pathological stimuli. Astrocyte activation is characterized by increased pro-inflammatory cytokines and oxidative stress. Given their role in neuroinflammation, targeting astrocytic mechanisms presents potential therapeutic opportunities. Ellagic acid (EA), a phenolic compound, exhibits antioxidant, anti-inflammatory, and neuroprotective effects. This study investigated the effect of EA on astrocytes from neonatal rats exposed to lipopolysaccharide (LPS), a potent proinflammatory agent widely used to induce neuroinflammation *in vitro*.

**Methods and results:**

Astrocytes were pretreated with EA (50, 100, and 200 µM) for 48 h and subsequently exposed to LPS (1 µg/mL) for 3h. Cell viability and proliferation, inflammatory and oxidative stress parameters were evaluated. LPS decreased astrocytic viability and increased cell proliferation and EA prevented these alterations. EA also attenuated the LPS-induced increase in reactive oxygen species and nitrite levels and the decrease in sulfhydryl content and antioxidant enzyme activities. LPS induced an increase in gene expression of interleukin 1β and tumor necrosis factor-alpha, and EA treatment attenuated these alterations. Conversely, EA increased interleukin 6 (IL-6) and glycogen synthase kinase-3 beta mRNA expression compared with LPS-only exposure. LPS increased IL-6 levels and decreased interleukin 10 (IL-10) levels in astrocytes, and EA at 100 µM prevented the reduction in IL-10 levels but did not significantly modify IL-6 levels compared with LPS group.

**Conclusion:**

EA modulates oxidative and inflammatory markers in LPS-stimulated astrocytes. Further studies are needed to clarify the underlying mechanisms and evaluate these effects in more complex models of neuroinflammation.

**Graphical abstract:**

**Figure:**
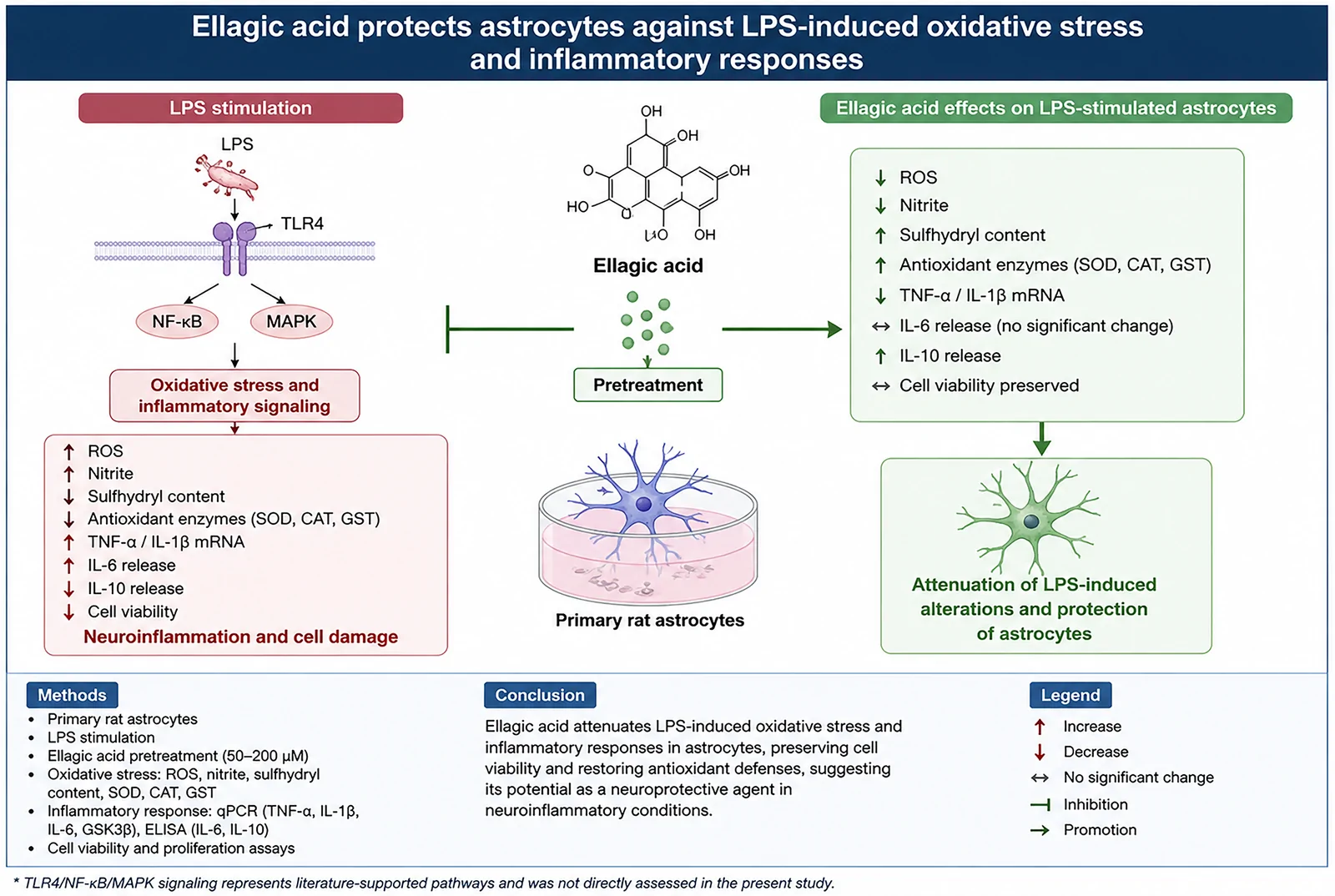

## Introduction

Ellagic acid (EA) is a phenolic compound found in various plant sources, including fruits such as pomegranates, strawberries, and raspberries, as well as certain herbs and seeds [1]. Its chemical structure comprises a hydrophilic portion containing four hydroxyl groups and two lactone groups, complemented by a lipophilic portion with two biphenyl rings [2]. In recent years, EA has garnered notable interest owing to its diverse pharmacological effects and extensive range of molecular targets [1]. Previous studies have highlighted the pivotal roles of its antioxidant and anti-inflammatory properties in contributing to its broad-spectrum pharmacological profile [3, 4]. Therefore, exploring the effects of EA on cellular processes associated with neuroinflammation may provide relevant insights into its potential biological actions in the central nervous system.

Neuroinflammation is a multifaceted process marked by the activation of glial cells in response to pathological stimuli such as injury, infection, or trauma within the nervous system [5]. Chronic neuroinflammation is recognized as a significant contributor to the progression and development of various neurological diseases such as Alzheimer’s disease, Parkinson’s disease, Huntington’s disease, and amyotrophic lateral sclerosis [6]. This process is orchestrated through the production of pro-inflammatory cytokines such as interleukin 1β (IL-1β), interleukin 6 (IL-6), and tumor necrosis factor-alpha (TNF-α), as well as reactive oxygen and nitrogen species by resident brain cells like microglia and astrocytes [5, 7].

Astrocytes play crucial and multifaceted roles in maintaining brain health and function [8, 9]. These glial cells have essential functions such as the maintenance of redox potential, metabolic support for neurons, and regulation of the extracellular environment, including ion balance and neurotransmitter levels [10]. Furthermore, astrocytes actively participate in immune responses within the central nervous system (CNS). Astrocyte reactivity is a hallmark of neuroinflammation and is characterized by cellular hypertrophy and the upregulation of glial fibrillary acidic protein (GFAP), an intermediate filament protein [10, 11]. Chronic astrogliosis can result in the sustained production of reactive oxygen and nitrogen species and the release of pro-inflammatory molecules such as IL-1β, IL-6, and TNF-α [11, 12]. In this context, astrocytes have the capacity to amplify or resolve neuroinflammatory responses, depending on the context and duration of the insult [10, 12]. Consequently, understanding the complex role of astrocytes in neuroinflammation may provide insights into potential strategies for modulating neuroinflammatory responses.

Lipopolysaccharide (LPS) is a well-known endotoxin derived from Gram-negative bacteria. It is commonly employed as a potent pro-inflammatory agent to induce inflammatory conditions in both in vitro and in vivo models within the field of neuroscience [13–16]. Previous studies have demonstrated that LPS induces alterations in cell viability, oxidative damage, and the release of inflammatory mediators in astrocytes [15, 16]. Consequently, LPS serves as a valuable research tool for investigating astrocyte responses and the effects of bioactive compounds under inflammatory conditions. Thus, this study aimed to investigate the effects of EA on cell viability, proliferation, oxidative stress, inflammatory cytokines expression and levels, and glycogen synthase kinase 3 beta (GSK3-β) mRNA expression in primary astrocyte cultures exposed to LPS. Based on the reported biological properties of EA, we hypothesized that this compound would attenuate LPS-induced astrocyte dysfunction by reducing oxidative stress and modulating the expression of inflammatory cytokines and GSK-3β.

## Materials and methods

### Chemicals and reagents

LPS from *Escherichia coli* (055:B5; cat. no. L2880), EA (cat. no. E2250), 4-(2-Hydroxyethyl) piperazine-1-ethane sulfonic acid (HEPES; cat. no. H3784), sodium bicarbonate (NaHCO_3_; cat. no. S5761), poly-L-lysine solution (cat. no. P4707), 3(4,5-dimethylthiazole-2-yl)-2,5 diphenyl tetrazolium bromide (MTT; cat. no. M5655), dimethylsulfoxide (DMSO; cat. no. D4540), sulforhodamine B (SRB; cat. no. S9012), TRIzol reagent (cat. no. T9424); dichloro-dihydro-fluorescein diacetate (DCFH-DA; cat. no. D6883), 1-chloro-2,4-dinitrobenzene (CDNB; cat. no. 237329) and 5,5′-dithiobis (2-nitrobenzoic acid) (DTNB; cat. no. D8130) were obtained from Sigma-Aldrich Co. (St. Louis, MO, USA). Trichloroacetic acid (TCA; cat. no. A1066) and hydrogen peroxide (H_2_O_2_; cat. no. 01P1013) were purchased from Synth^®^ in Brazil. Dulbecco’s modified Eagle’s medium (DMEM; cat. no. 31600034), fungizone (cat. no. 15290018), penicillin/streptomycin (cat. no. 15070063), 0.5% trypsin/ethylene diamine tetraacetic acid (EDTA) solution (cat. no. 15400054), and fetal bovine serum (FBS; cat no. 12657029) were obtained from Gibco in Carlsbad, CA, USA. All other reagents used in the experiments were of analytical grade and highest purity available.

### Animals

All animal procedures were approved by the Committee of Ethics and Animal Experimentation of the Federal University of Pelotas, Brazil under protocol number CEEA 027159/2022-4, and were conducted in accordance with the Brazilian Guidelines for the Care and Use of Animals in Scientific Research Activities (DCBA 2013) and the National Council of Control of Animal Experimentation (CONCEA).

### Astrocyte culture

Primary astrocyte cultures were prepared as previously described [16]. Newborn Wistar rats (1–2 days old, males or females) were euthanized by decapitation, and the brains were removed. The cerebral cortex was isolated and mechanically dissociated in calcium- and magnesium-free balanced salt solution (pH 7.4) to form a cell suspension. Cerebral cortical tissue from three neonatal rats was used for culture preparation, and the resulting cell suspensions were pooled before further processing. The pooled cell suspension was centrifuged at 1000 rpm for 10 min, and the pellet was resuspended in DMEM supplemented with 10% FBS (pH 7.6). Cells were seeded on poly-L-lysine-coated plates at different densities according to the experimental procedure: 3 × 10⁴ cells/well in 96-well plates for cytotoxicity assays, 3 × 10⁵ cells/well in 6-well plates for oxidative stress assays, and 8 × 10⁵ cells/well in 6-well plates for qPCR and cytokine measurements. Cell cultures were maintained at 37 °C in a humidified atmosphere containing 5% CO₂ for 20 days to allow cell maturation and differentiation, with the culture medium changed every 4 days. No additional purification procedure or immunocytochemical assessment of culture purity was performed. Therefore, although the cultures were prepared according to a standardized protocol for primary astrocyte culture [16], the presence of other glial cell types cannot be excluded.

### Treatment with LPS and EA

**Fig. 1:**
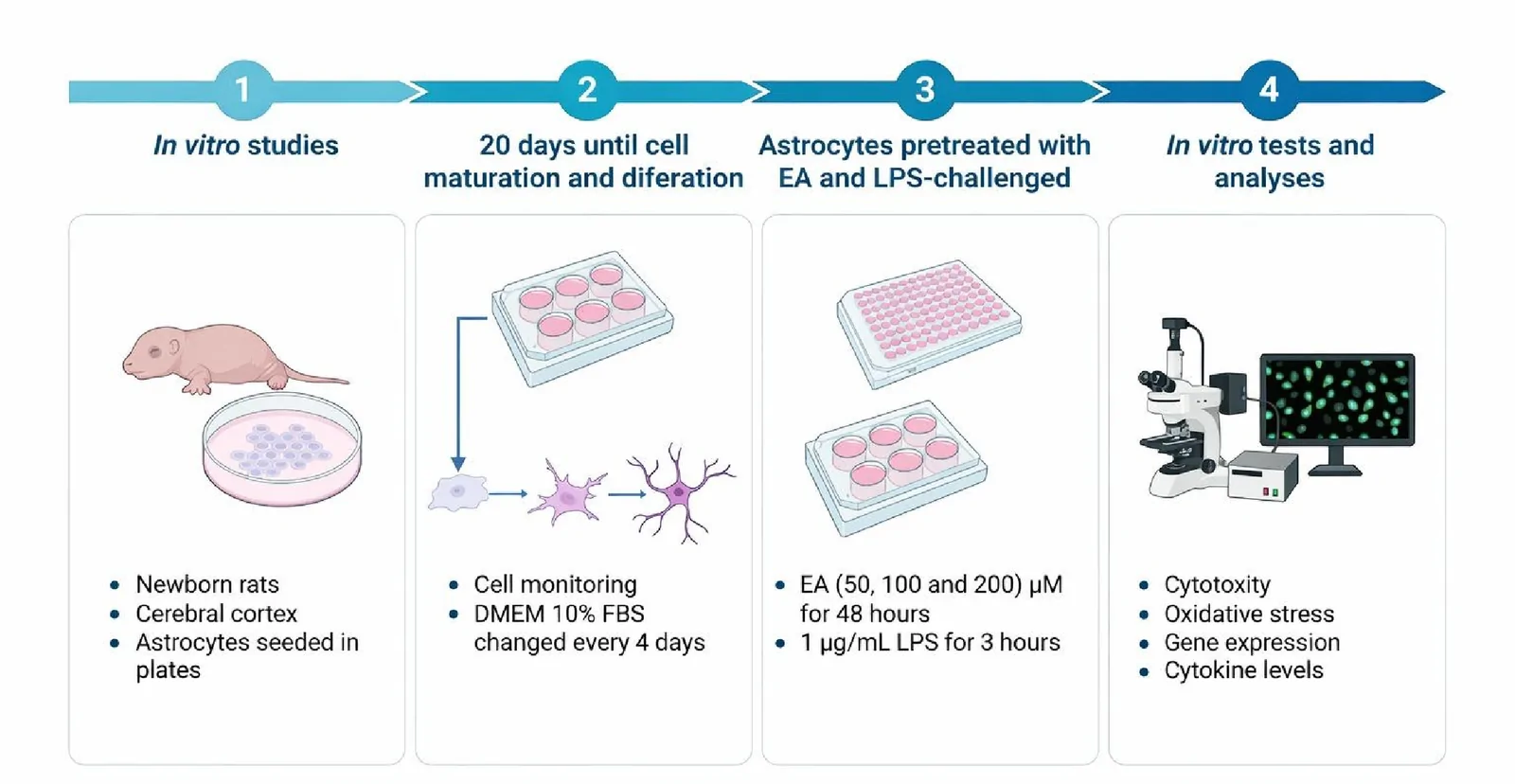
Experimental scheme of ellagic acid (EA) treatment in primary astrocytes culture and exposure to lipopolysaccharide (LPS).

The primary astrocyte cultures were initially exposed to EA at different concentrations (50, 100, and 200 µM) for 48 h. Subsequently, cells were exposed to 1 µg/mL LPS for 3 h (Fig. 1). EA was dissolved in DMSO and subsequently diluted in DMEM to obtain the final concentrations of 50, 100, and 200 µM. The EA preparations were homogenized by vortexing for approximately 30 s immediately before use. No visible precipitation was observed in the EA preparations, including at the highest concentration tested (200 µM). No filtration or sonication was performed. The final DMSO concentration in the culture medium was maintained below 1%, and control cells received the same final concentration of DMSO as the corresponding EA-treated groups. The experimental protocol and the duration of exposure to LPS were consistent with previous studies conducted by our research group using astrocyte cultures [15, 16].

### Cell viability

Cell viability was determined using the MTT assay, which consists of measuring the number of cells with metabolically active mitochondria based on the reduction of the tetrazolium salt MTT to formazan [17]. The cultures were washed with CMF and then incubated with MTT solution (0.5 mg/mL per well) at 37 °C in a humidified 5% CO₂ atmosphere for 90 min. After incubation, the medium was removed, and the formazan crystals were dissolved in DMSO. Finally, optical density (OD) was measured at 492 nm using a microplate reader. Results were calculated using Prism 8.0 software (Prism GraphPad Software, San Diego, USA) and expressed as percentage of the control using the following formula: Cell viability rate (%) = (OD of treated cells/OD of control) × 100%.

### Cell proliferation

The SRB assay was used to determine cell proliferation based on the measurement of cell protein [18]. Initially, cultures were washed and fixed in 50% TCA for 45 min at 4 °C. After this period, five washes were performed with distilled water. Subsequently, 0.4% SRB was added and incubated in the dark at room temperature for 30 min. Afterward, the cells were washed five times with 1% acetic acid to completely remove unbound dye, and SRB was eluted with 10 mM Tris. OD was measured at 530 nm using a microplate reader, and the results were calculated using Prism 8.0 software (Prism GraphPad Software, San Diego, USA) according to the following formula: Cell proliferation rate (%) = (OD of treated cells/OD of control) × 100%.

### Oxidative stress analysis

After 48 h of treatment, cultures were washed twice with sterile water, and the lysate was manually prepared with the aid of a cell scraper. Then, the supernatant was centrifuged at 1000 rpm for 10 min. The pellet was discarded, and the supernatant was used for oxidative stress analyses. Protein quantification was conducted as previously described [19], using bovine serum albumin as the standard.

#### Reactive oxygen species (ROS) assay

ROS levels were evaluated according to previously described methods with some modifications [16, 20]. This method is based on the reaction of DCFH-DA with intracellular ROS to generate a fluorescent intermediate. Intact cells were incubated with 1 µM DCFH-DA for 30 min at 37 °C. After incubation, cells were washed with PBS, and the fluorescence intensity was measured using a multi-well plate reader (485/520 nm). ROS production was expressed as µmol DCF/mg protein.

#### Nitrite levels assay

Nitrite levels were evaluated in cell supernatants according to the method of Stuehr and Nathan [21], using a colorimetric reaction with Griess reagent. Initially, 5% sulfanilamide in phosphoric acid was added to the cell culture supernatants. The mixture was then incubated at room temperature for 10 min. Subsequently, Griess reagent (0.1% N-[1-naphthyl] ethylenediamine dihydrochloride) was added to the samples and incubated for an additional 10 min in the dark. The absorbance was measured at 540 nm, and nitrite levels were determined using a standard curve of sodium nitrite.

#### Total sulfhydryl (SH) levels assay

The total SH content was determined in astrocyte lysates using the DTNB method, following the protocol described by Aksenov and Markesbery [22]. This assay relies on the reduction of DTNB by thiols, resulting in the generation of the yellow derivative 2-nitro-5-thiobenzoic acid (TNB). In brief, the samples were added to PBS containing EDTA (pH 7.4), and the reaction was initiated by the addition of DTNB. The absorbance was measured using a microplate reader at 412 nm. The results were expressed as nmol TNB/mg protein.

#### Superoxide dismutase (SOD) activity

SOD activity was analyzed in cell lysates according to the method of Misra and Fridovich [23]. This method is based on the inhibition of adrenaline auto-oxidation through the elimination of superoxide anions by SOD. Absorbance was recorded kinetically at 480 nm, and SOD activity was expressed as units/mg protein.

#### Catalase (CAT) activity

CAT activity was measured using a previously described method [24]. This method is based on the decomposition of H₂O₂ in a potassium phosphate buffer (pH 7.0). The decrease in absorbance resulting from H₂O₂ decomposition was monitored kinetically at 240 nm. CAT activity was expressed as units/mg protein.

#### Glutathione S-transferase (GST) activity

GST activity was assessed following the method described by Habig et al. [25]. This method uses CDNB as a substrate. The reaction mixture contained 1 mM CDNB diluted in ethanol, 10 mM glutathione (GSH), 20 mM potassium phosphate buffer (pH 6.5), and 20 µL of cell lysate. The formation of the GS-DNB conjugate was monitored kinetically at 340 nm. GST activity was expressed as µmol GS-DNB/min/mg protein.

### RNA extraction, cDNA synthesis and quantitative real-time polymerase chain reaction for IL-1β, IL-6, TNF-α and GSK3-β

Total mRNA was extracted from astrocytes using TRIzol reagent (Invitrogen™, Carlsbad, USA) followed by DNase treatment with DNase I Amplification Grade (Invitrogen™, Carlsbad, USA) to minimize DNA contamination of the samples. The isolated total RNA was quantified and its purity (260/280 and 260/230 ratios) was examined using a NanoVue spectrophotometer (GE, Fairfield, CT, USA).

The cDNA synthesis was performed using the High-Capacity cDNA Reverse Transcription kit (AppliedBiosystems™, UK) according to the manufacturer’s protocol. For reverse transcription, 500 ng of total RNA was used in a reaction volume of 20 µL. Amplification was performed using GoTaq^®^ qPCR Master Mix (Promega, Madison, WI, USA) and the QuantStudio 3 Real-Time PCR System (ThermoFisher, USA). Primer sequences were obtained from previously published studies [26–30] and used for gene expression analysis in primary rat astrocyte cultures. The corresponding GenBank accession numbers were added to Table 1 to enable identification of the reference sequences targeted by each primer pair. The qPCR conditions were as follows: 10 min at 95 °C to activate the hot-start Taq polymerase, followed by 35 cycles of denaturation for 15 s at 95 °C, primer annealing for 60 s at 60 °C, and extension for 30 s at 72 °C (fluorescence signals were detected at the end of every cycle). Baseline and threshold values were automatically set by the QuantStudio Design Analysis software.

**Table 1:** Primers used for quantitative real-time polymerase chain reaction. Listed are the forward and reverse primer sequences used to amplify each target gene and the β‑actin endogenous control

Primer Name^a^	Sequence	Reference
β‑actin Forward	5’ ACCCGCGAGTACAACCTTCT 3’	Solgi et al. [26]
β‑actin Reverse	5’ ATACCCACCATCACACCCTGG 3’	
IL-6 Forward	5’ TGAAGAACAACTTACAAGATAAC 3’	Dupius et al. [27]
IL-6 Reverse	5’ CATTAGGAGAGCATTGGAA 3’	
IL-1β Forward	5’ GACAGAACAATAAGCCAACA 3’	Aires et al. [28]
IL-1β Reverse	5’ ACACAGGACAGGTATAGATT 3’	
GSK3-β Forward	5’ TACCCATACGATGTTCCAGAT 3’	Jiang et al. [29]
GSK3- β Reverse	5’ ACCCTGCCCAGGAGTTGCCAC 3’	
TNF-α Forward	5’ TCAGCCGATTTGCCATTTCAT 3’	Cai et al. [30]
TNF-α Reverse	5’ ACACGCCAGTCGCTTCACAGA 3’	

^a^Genbank acession numbers: NM_031144.3 (β‑actin), NM_031168.2 (IL-6), NM_008361.4 (IL-1β), NM_019827.7 (GSK3-β), and NM_013693.3 (TNF- α)

The number of PCR cycles required to reach the fluorescence threshold in each sample was defined as the Ct value. Five replicates were analyzed for each experimental group. The 2^−ΔΔCt^ method was used to determine relative gene expression [31], using β-actin as the housekeeping gene.

### Determination of IL-6 and IL-10 levels

IL-6 and IL-10 levels were quantified using an enzyme-linked immunosorbent assay kit (RAB0311 and RAB0246, Sigma-Aldrich, St. Louis, MO, USA) according to the manufacturer’s instructions. The results were expressed as pg/mL.

### Statistical Analysis

Data normality was assessed using the Shapiro–Wilk test, and homogeneity of variances was assessed using the Brown–Forsythe test. Differences between groups were analyzed using one-way ANOVA followed by Tukey’s multiple comparisons test in GraphPad Prism 8 (GraphPad Software, USA). The results were expressed as mean ± SEM, and statistical significance was set at *P* < 0.05.

## Results

### EA prevents LPS-induced damage to astrocytes viability and proliferation

**Fig. 2:**
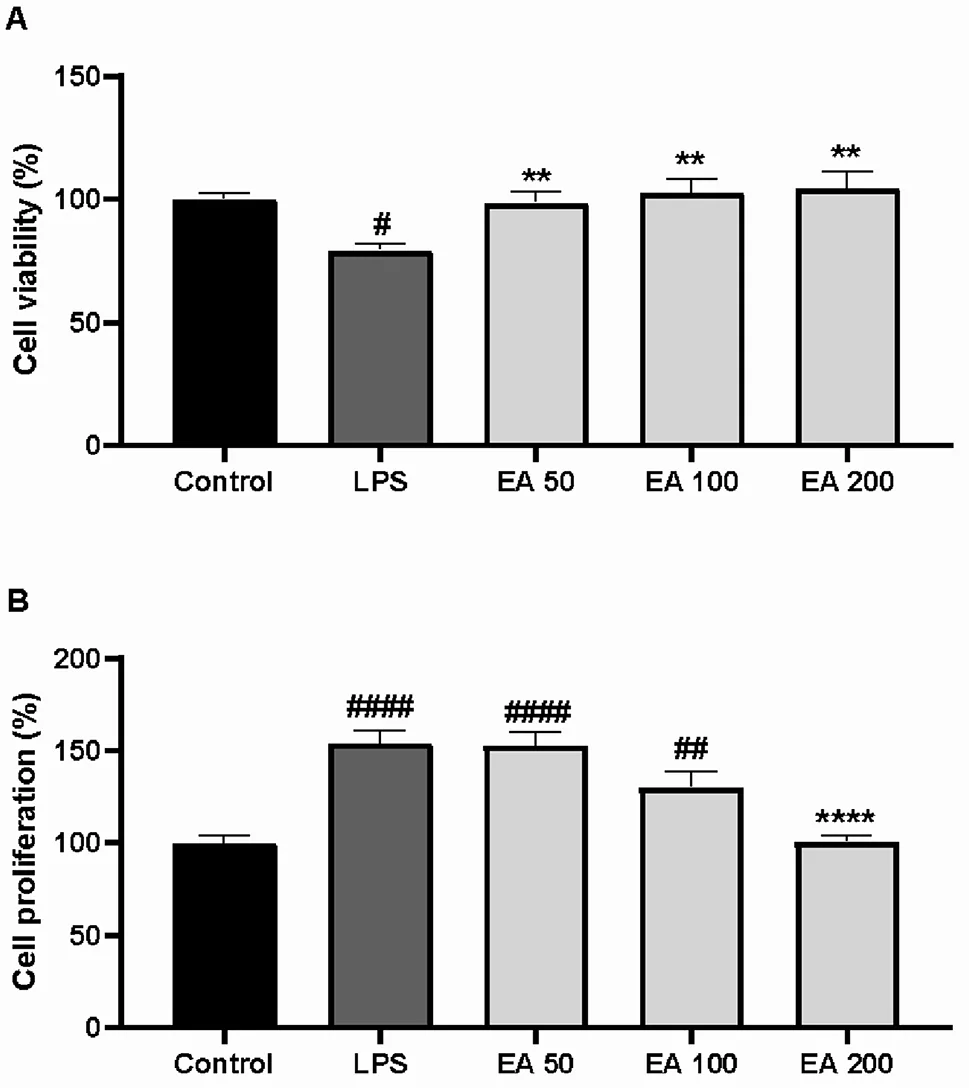
Cellular viability and proliferation of astrocytes treated with ellagic acid (EA) and exposed to lipopolysaccharide (LPS). The values represent the mean ± standard error of the mean (SEM). MTT and SRB assays were performed using four technical replicates per experimental condition, with cells derived from the pooled primary astrocyte culture. Data analysis was performed using one-way analysis of variance followed by Tukey’s post hoc test. **P <0.01 and ****P< 0.0001 indicates a significant difference compared with LPS group. ^#^*P*< 0.05, ^##^*P*< 0.01, and ^####^*P*<0.0001 indicates a significant difference compared with control cells.

A one-way ANOVA showed a significant effect of treatment on astrocyte viability (F(4,55) = 4.88, *P* = 0.0019). LPS exposure significantly decreased astrocytes viability compared with control cells (*P* = 0.0190). In contrast, EA treatment at all concentrations significantly increased cell viability compared with the LPS-exposed group (EA 50, 100, and 200 µM: *P* = 0.0320, 0.0064, and 0.0025, respectively) (Fig. 2A). A one-way ANOVA also showed a significant effect of treatment on astrocytes proliferation (F(4,55) = 18.42, *P* < 0.0001). Furthermore, LPS exposure increased cell proliferation after 48 h (*P* < 0.0001), an effect attenuated by EA treatment at 200 µM (*P* < 0.0001) (Fig. 2B).

### EA prevents oxidative damage caused by LPS

**Fig. 3:**
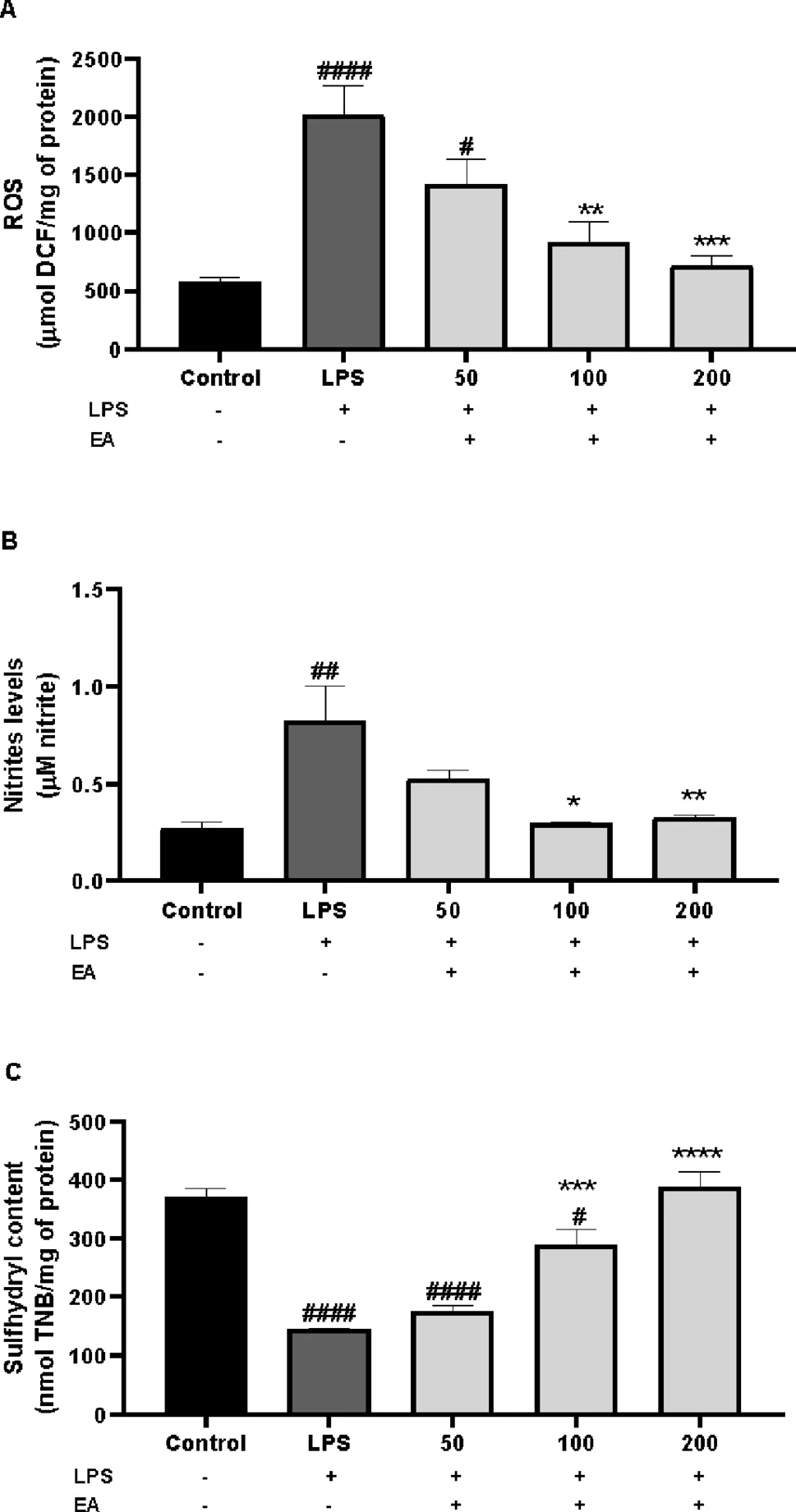
Reactive oxygen species (ROS) (A), nitrites (B), and total sulfhydryl (SH) (C) in astrocytes treated with ellagic acid (EA) and exposed to lipopolysaccharide (LPS). The values represent the mean ± standard error of the mean (SEM). ROS, nitrite, and SH assays were performed using two technical replicates per experimental condition, with cells derived from the pooled primary astrocyte culture. Data analysis was performed using one-way analysis of variance followed by Tukey’s post hoc test. *P < 0.05, **P < 0.01, ***P < 0.001 and ****P < 0.0001 indicates a significant difference compared with LPS group. ^#^*P* < 0.05, ^##^*P* < 0.01, and ^####^*P* < 0.0001 indicates a significant difference compared to control cells.

A one-way ANOVA showed significant effects of treatment on ROS (F(4,55) = 10.93, *P* < 0.0001), nitrite (F(4,55) = 6.57, *P* = 0.0012), and SH levels (F(4,55) = 34.60, *P* < 0.0001). LPS exposure significantly increased ROS (*P* < 0.0001) and nitrite levels (*P* = 0.0017) and decreased SH content (*P* < 0.0001) compared with the control group (Fig. 3). However, EA treatment significantly attenuated the LPS-induced increase in ROS at 100 and 200 µM (*P* = 0.0017 and *P* = 0.0002, respectively) and in nitrite levels at the same concentrations (*P* = 0.0175 and *P* = 0.0049, respectively). Furthermore, EA significantly increased SH levels at concentrations of 100 µM (*P* = 0.0001) and 200 µM (*P* < 0.0001) compared with the LPS-exposed group (Fig. 3).

### Effect of EA on antioxidant enzyme activity in astrocytes exposed to LPS

**Fig. 4:**
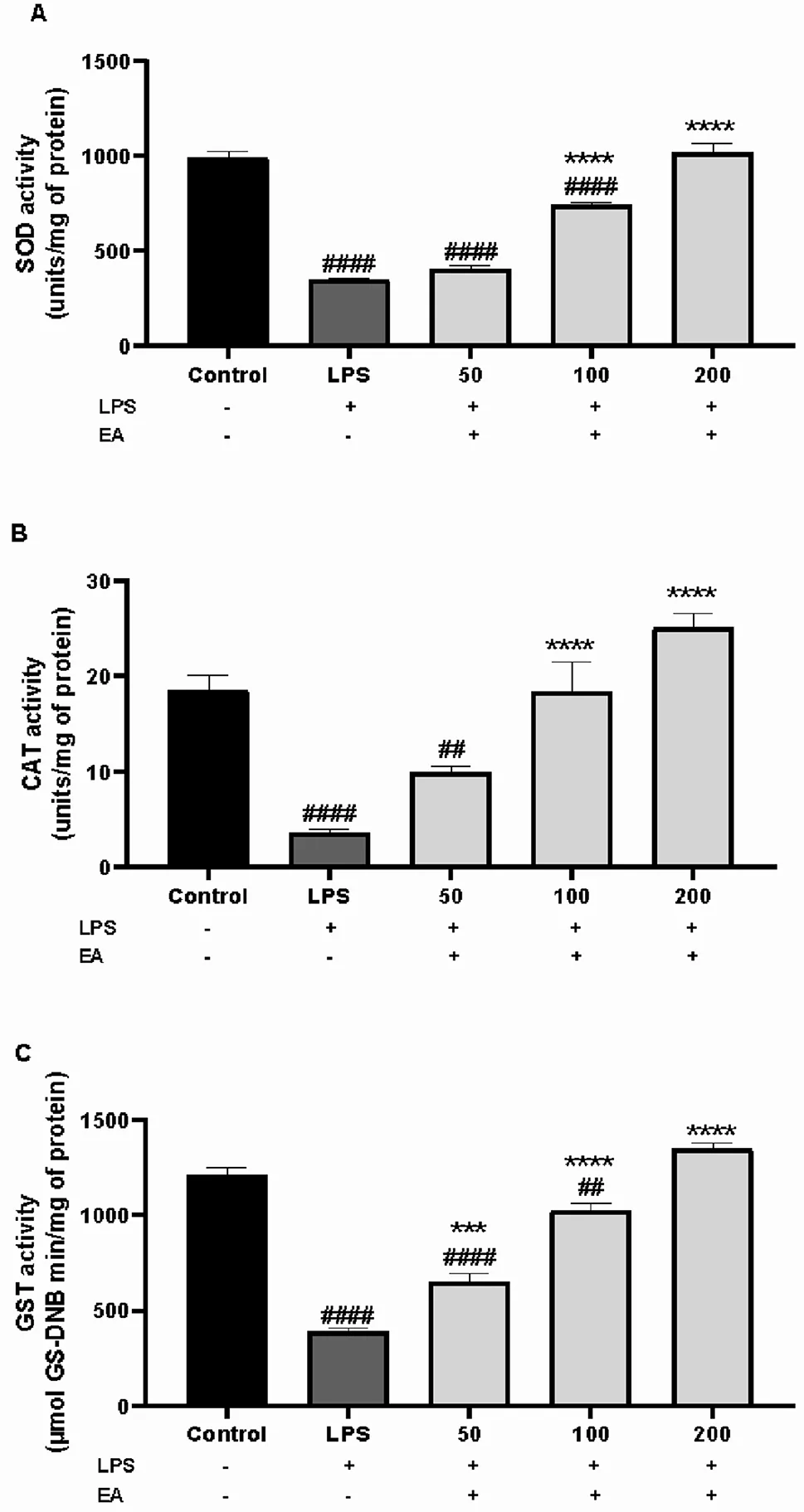
Superoxide dismutase (**A**), catalase (**B**), and glutathione S-transferase (**C**) activities in astrocytes treated with ellagic acid (EA) and exposed to lipopolysaccharide (LPS). The values represent the mean ± standard error of the mean (SEM). SOD, CAT, and GST assays were performed using two technical replicates per experimental condition, with cells derived from the pooled primary astrocyte culture. Data analysis was performed using one-way analysis of variance followed by Tukey’s post hoc test. ***P < 0.001 and ****P <0.0001 indicates a significant difference compared with LPS group. ^##^*P* <0.01 and ^####^*P* < 0.0001 indicate a significant difference compared with control cells.

The findings regarding the activities of antioxidant enzymes are shown in Fig. 4. A one-way ANOVA showed significant effects of treatment on SOD activity (F(4,55) = 118.1, *P* < 0.0001), CAT activity (F(4,55) = 24.96, *P* < 0.0001), and GST activity (F(4,55) = 127.0, *P* < 0.0001). LPS exposure significantly decreased SOD, CAT, and GST activities (*P* < 0.0001 for all comparisons). However, EA treatment attenuated these changes. EA at 100 and 200 µM significantly increased SOD and CAT activities compared with the LPS-exposed group (*P* < 0.0001). Furthermore, GST activity was significantly increased by EA at 50 µM (*P* = 0.0002), 100 µM (*P* < 0.0001), and 200 µM (*P* < 0.0001) compared with the LPS-exposed group.

### Effect of EA on TNF-α, IL-1β, IL-6, and GSK3-β mRNA expression in astrocytes exposed to LPS

**Fig. 5:**
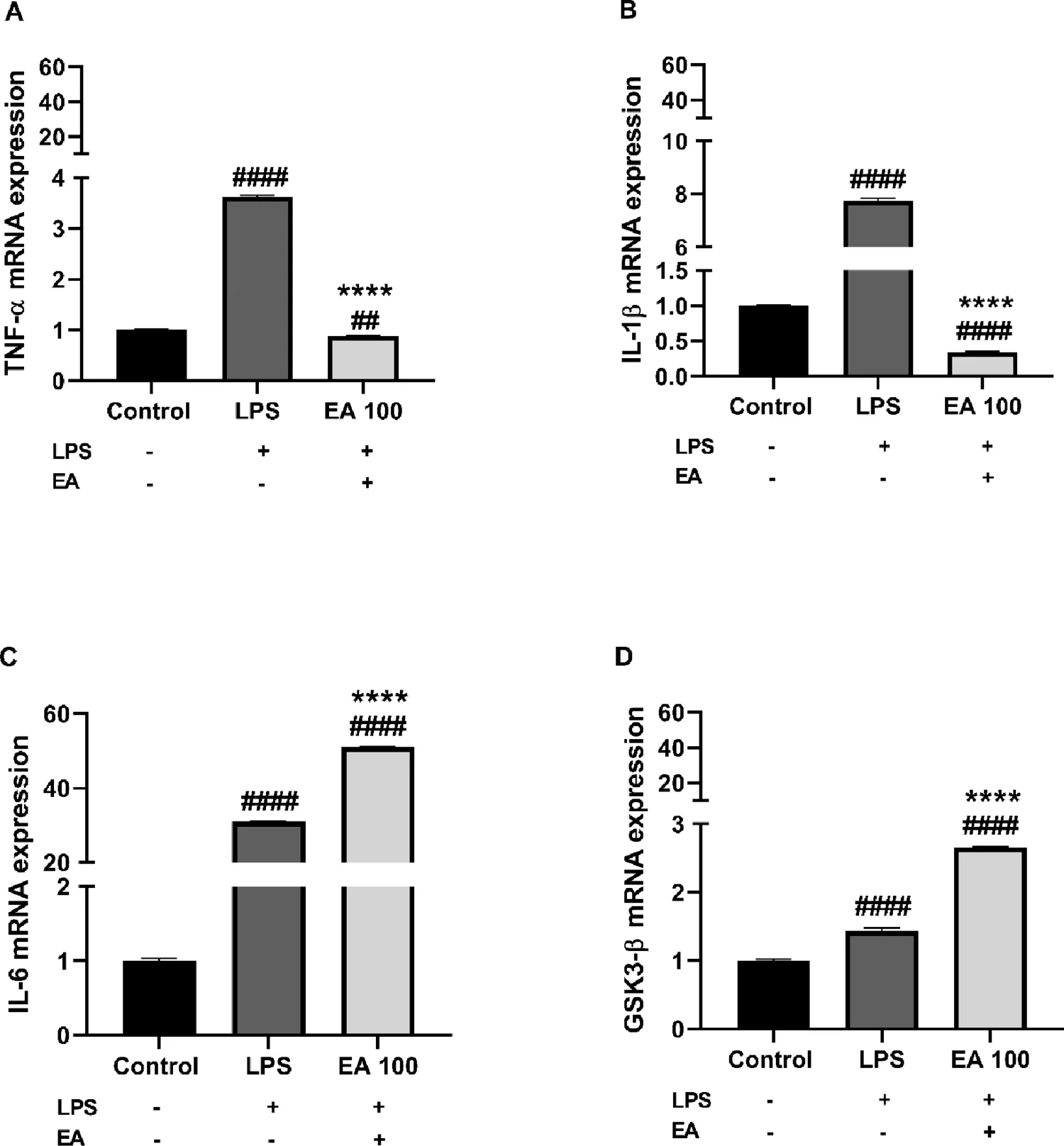
Gene expression of IL-1β, TNF-α, IL-6, and GSK3β in astrocytes treated with ellagic acid (EA) and exposed to lipopolysaccharide (LPS). Values are presented as mean ± standard error of the mean (SEM), with five technical replicates analyzed per experimental group. Data were analyzed by one-way analysis of variance followed by Tukey’s post hoc test. ****P <0.0001 indicates a significant difference compared with LPS group. ^##^*P*< 0.01 and ^####^*P*< 0.0001 indicates a significant difference compared with control cells.

A one-way ANOVA showed significant effects of treatment on TNF-α (F(2,12) = 6696, *P* < 0.0001), IL-1β (F(2,12) = 4433, *P* < 0.0001), IL-6 (F(2,12) = 50213, *P* < 0.0001), and GSK3-β mRNA expression (F(2,12) = 1211, *P* < 0.0001). The relative mRNA expression levels of TNF-α, IL-1β, IL-6, and GSK3-β were evaluated in astrocyte cultures following EA pretreatment and LPS exposure (Fig. 5). The relative mRNA expression of all genes was increased in astrocyte cultures exposed to LPS compared with untreated cells (*P* < 0.0001). However, treatment with EA (100 µM) prevented the LPS-induced increase in TNF-α and IL-1β mRNA expression (*P* < 0.0001) (Fig. 5A and B). In contrast, EA significantly increased IL-6 and GSK3-β mRNA expression compared with cells exposed to LPS (*P* < 0.0001) (Fig. 5C and D).

### Effect of EA on IL-6 and IL-10 releases in astrocytes exposed to LPS

**Fig. 6:**
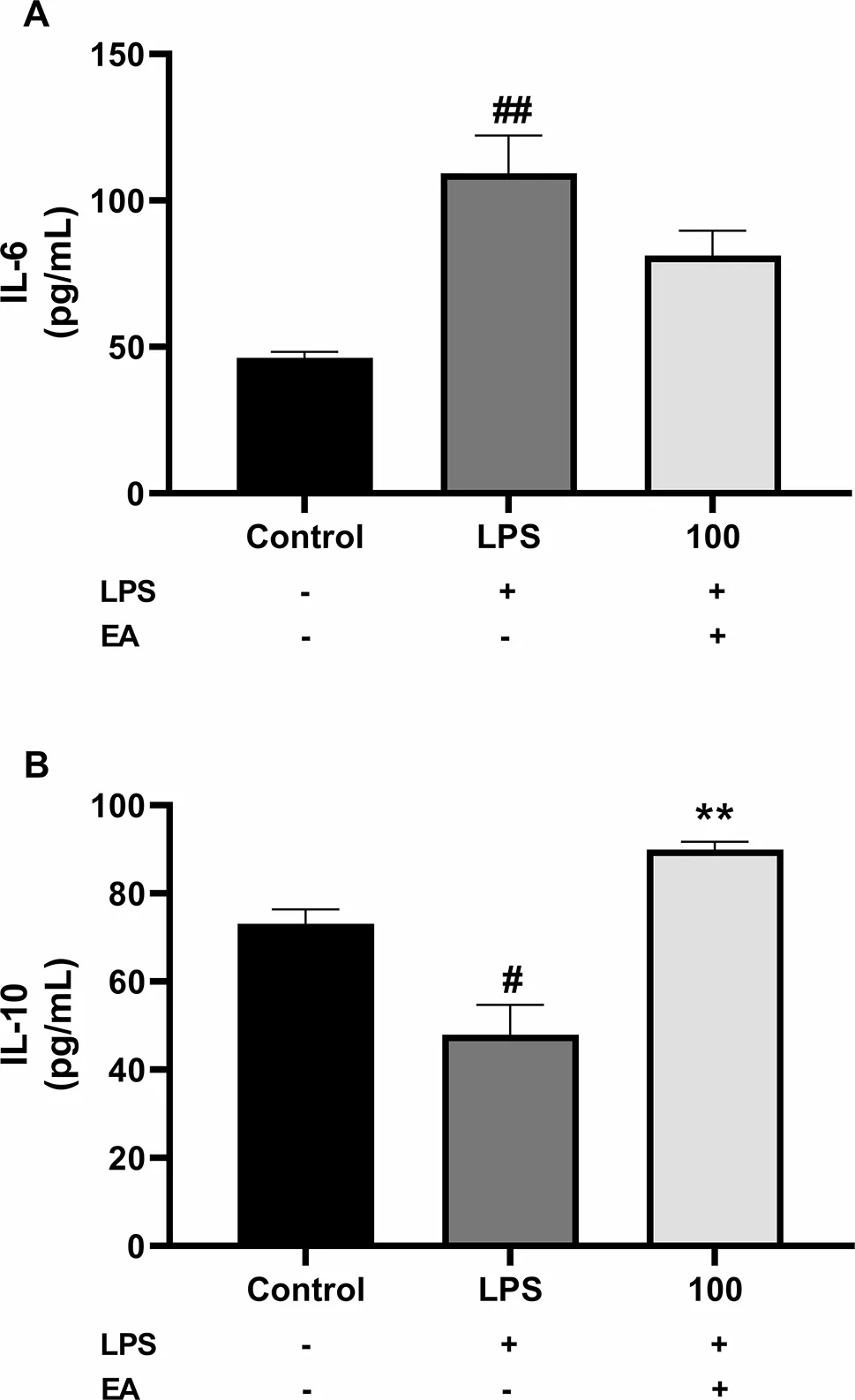
IL-6 and IL-10 extracellular levels in astrocytes treated with ellagic acid (EA) and exposed to lipopolysaccharide (LPS). Values represent the mean ± standard error of the mean (SEM). IL-6 and IL-10 measurements were performed using three technical replicates per experimental condition, with cells derived from the pooled primary astrocyte culture. *P< 0.05 and **P< 0.01 indicates a significant difference compared with LPS group. ^##^*P*< 0.01 indicates a significant difference compared with control cells.

A one-way ANOVA showed significant effects of treatment on IL-6 (F(2,6) = 12.29, *P* = 0.0075) and IL-10 levels (F(2,6) = 22.58, *P* = 0.0016). LPS exposure significantly increased IL-6 release compared with control cells (*P* = 0.0062) (Fig. 6A). EA at 100 µM did not significantly modify extracellular IL-6 levels compared with the LPS-exposed group. LPS also significantly reduced IL-10 levels compared with control cells (*P* = 0.0167), whereas treatment with EA at 100 µM prevented this reduction (*P* = 0.0013) (Fig. 6B).

## Discussion

The present study demonstrates that EA exerts an antioxidant effect and modulates inflammation-associated parameters in primary astrocytes exposed to LPS. Consistent with previous studies, exposure to LPS resulted in significant changes in astrocyte viability and proliferation, accompanied by oxidative stress and alterations in pro-inflammatory markers [15, 16].

LPS effects are mediated by the activation of Toll-like receptor 4 (TLR4) located on the cell membrane of astrocytes. This receptor can recognize LPS as a pathogen-associated molecular pattern (PAMP), and after activation by LPS, astrocytic TLR4 initiates an intracellular signaling cascade, leading to a significant increase in the expression of apoptotic factors, autophagy-related components, and several inflammatory mediators, including IL-1β, IL-6, and TNF-α [32, 33]. Furthermore, oxidative damage is exacerbated by mitochondrial dysfunction, with increased levels of reactive oxygen and nitrogen species contributing to the amplification of the inflammatory response [34].

In the present study, we observed that EA effectively mitigated the alterations induced by LPS in cell viability and proliferation in primary astrocyte cultures. The apparent discrepancy between the MTT and SRB results may reflect the different biological parameters assessed by these assays. While the MTT assay reflects cellular metabolic activity, the SRB assay estimates cellular protein content and biomass [17]. Thus, the MTT results together with increased SRB staining following LPS exposure may reflect distinct metabolic and cellular responses to the inflammatory stimulus. However, a cell-free control containing EA and MTT was not performed. Therefore, a potential direct interaction between EA and the MTT assay cannot be excluded, particularly because polyphenolic compounds may interfere with MTT reduction.

EA treatment also attenuated the LPS-induced increase in ROS and nitrite levels and increased antioxidant enzyme activities, contributing to the maintenance of redox homeostasis in astrocytes. Consistent with our findings, other studies have also demonstrated the potential of EA to prevent oxidative damage induced by cadmium in astrocytes [35], reduce LPS-induced nitrite production in macrophages [36], and alleviate mitochondrial dysfunction and oxidative damage in cardiomyocytes exposed to clozapine [37]. Thus, the present findings suggest that EA may modulate astrocytic redox homeostasis under inflammatory conditions.

EA is composed of two lactone groups and four hydroxyl groups, rendering it effective in neutralizing a broad spectrum of reactive oxygen and nitrogen species [38, 39]. The antioxidant capacity of EA can be attributed to its ability to scavenge free radicals such as hydroxyl radicals and peroxynitrite [38, 39]. Additionally, its capacity for metal chelation also contributes to the reduction of free radical production, thus providing protection against oxidative stress [38]. In the present study, we also demonstrated that EA caused an upregulation in the activities of key antioxidant enzymes such as SOD, CAT, and GST. These results are consistent with other studies in the literature where EA administration has been shown to positively influence the activity of SOD and CAT enzymes in a preclinical model of Parkinson’s disease [40]. Taken together, these different mechanisms involved in the antioxidant action of EA may contribute to the decrease in levels of ROS and nitrites observed in astrocytes exposed to LPS.

The antioxidant effects of EA may also involve the modulation of endogenous antioxidant pathways. Among these, the nuclear factor erythroid 2-related factor 2 (Nrf2) signaling pathway has been recognized as a central regulator of the cellular antioxidant response by controlling the expression of antioxidant enzymes such as SOD, CAT, and GST [41–43]. In a previous study, EA was shown to attenuate oxidative damage through modulation of the Nrf2 pathway [42]. However, Nrf2 expression or activation was not evaluated in the present study. Therefore, although previous evidence supports Nrf2 as a biologically plausible pathway involved in the antioxidant actions of EA, its contribution to the effects observed in LPS-stimulated astrocytes cannot be established from the present data. Future studies directly assessing Nrf2 expression and activation will be required to investigate its potential involvement.

Indeed, alterations in redox balance have been consistently associated with LPS-triggered inflammatory processes. This imbalance between levels of reactive species and antioxidant defense systems can lead to the oxidation of lipids, proteins, and nucleic acids, triggering a cascade of exacerbated inflammatory events in astrocytes [44]. Furthermore, redox-sensitive signaling pathways, including mitogen-activated protein kinases (MAPK) and nuclear factor kappa B (NF-κB), have been extensively described as important regulators of inflammatory responses induced by LPS, contributing to the expression of cytokines such as IL-1β, IL-6, and TNF-α [45]. Consistent with these inflammatory effects of LPS, we observed increased IL-1β and TNF-α mRNA expression and increased extracellular IL-6 levels following LPS exposure.

In this study, EA effectively prevented the LPS-induced increase in TNF-α and IL-1β mRNA expression. Previous studies have suggested that EA may attenuate the expression of pro-inflammatory cytokines through modulation of the NF-κB pathway [46, 47]. When astrocytes are exposed to pathogenic stimuli such as LPS, NF-κB is activated, and its nuclear translocation induces the transcription of genes related to inflammation, leading to the production of inflammatory mediators such as the cytokines IL-1β and TNF-α [46]. Previous studies have suggested that EA may attenuate inflammatory responses through modulation of NF-κB signaling, including effects on IκB phosphorylation and NF-κB nuclear translocation [46, 47]. Although modulation of NF-κB signaling could contribute to the reduction in TNF-α and IL-1β mRNA expression observed in the present study, NF-κB activation was not directly assessed in our experimental model. Therefore, the involvement of this pathway cannot be established from the present data, and additional studies directly assessing NF-κB activation are required.

Interestingly, EA treatment increased GSK3-β mRNA expression in astrocytes exposed to LPS. GSK3-β is a multifunctional kinase involved in several cellular processes, including the regulation of inflammatory responses. Beurel and Jope [48] demonstrated that GSK3-β activity positively regulates IL-6 production in glial cells, suggesting that, under specific conditions, increased GSK3-β signaling may contribute to inflammatory responses. However, it is important to emphasize that GSK3-β activity is primarily regulated by post-translational mechanisms, particularly phosphorylation status, rather than transcriptional expression alone. Therefore, the increased GSK3-β mRNA observed in the present study does not necessarily indicate increased kinase activity or enhanced inflammatory signaling. Further studies evaluating GSK3-β protein expression, phosphorylation status, and downstream signaling pathways are necessary to clarify the functional significance of this finding in EA-treated astrocytes.

Additionally, although EA increased IL-6 mRNA expression, this transcriptional response was not accompanied by a corresponding increase in extracellular IL-6 levels measured in culture supernatants. This apparent discrepancy may reflect the complex regulation of cytokine production, which involves multiple regulatory steps beyond gene transcription, including mRNA stability, translational efficiency, intracellular processing, and secretion. Therefore, alterations in IL-6 mRNA expression do not necessarily result in proportional changes in extracellular cytokine levels, particularly when evaluated at a single experimental time point, as transcriptional activation and cytokine release may follow distinct temporal patterns [49, 50].

IL-6 is a pleiotropic cytokine whose biological effects are highly dependent on the cellular context, inflammatory stimulus, duration of activation, and the balance between classical and trans-signaling pathways [49]. Therefore, the increase in IL-6 mRNA observed after EA treatment should not be interpreted in isolation as evidence of either enhanced pro-inflammatory activity or an anti-inflammatory response. Instead, when considered together with the reduction in TNF-α and IL-1β mRNA expression, restoration of IL-10 levels, attenuation of oxidative stress, and preservation of astrocyte viability, the present findings suggest that EA selectively modulates astrocytic responses to LPS rather than causing a generalized suppression of inflammatory signaling.

Several limitations should be considered when interpreting the present findings. First, although primary astrocyte cultures provide a useful experimental model for investigating astrocyte responses, they do not fully recapitulate the cellular complexity of the central nervous system. Furthermore, because culture purity was not directly assessed, the presence and potential contribution of other glial cell types cannot be completely excluded. Second, EA-only groups were not included, limiting the ability to distinguish the effects of EA itself from its modulation of LPS-induced responses, particularly regarding the increased IL-6 and GSK3-β mRNA expression. Third, the EA concentrations used under these *in vitro* conditions may not directly correspond to clinically achievable concentrations because of its limited bioavailability and pharmacokinetic properties. Therefore, the findings should be interpreted within the context of an *in vitro* experimental model rather than as evidence of therapeutic efficacy in humans. Finally, all analyses were performed at a single early time point (3h) after LPS stimulation, and TNF-α, IL-1β, and GSK3-β were evaluated only at the transcriptional level. Moreover, signaling pathways potentially involved in EA activity, including NF-κB, Nrf2, MAPK, and GSK3-β signaling, were not directly assessed. Future studies incorporating time-course analyses, protein and phosphorylation measurements, and functional approaches are needed to further characterize these responses.

**Fig. 7:**
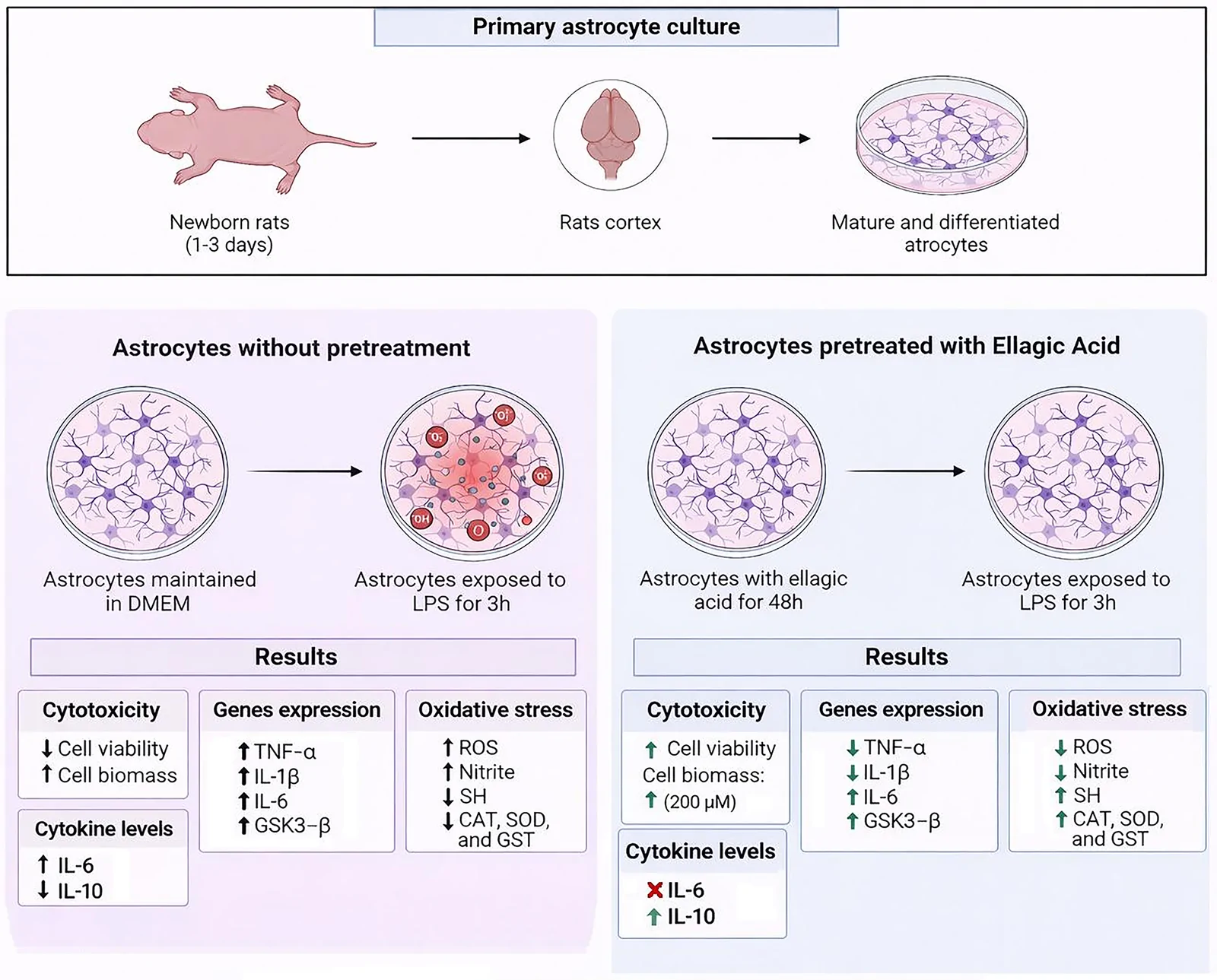
Overview of the main findings. Ellagic acid (EA) attenuated LPS-induced alterations in cell viability, oxidative stress, and inflammation-associated markers in primary astrocyte cultures. These findings indicate that EA modulates astrocytic responses under inflammatory conditions and provide insights into its biological effects in an in vitro model of neuroinflammation.

In summary, EA demonstrated antioxidant activity and modulated inflammation-associated responses in LPS-stimulated astrocytes. By attenuating the expression of selected pro-inflammatory markers, restoring IL-10 levels, and strengthening endogenous antioxidant defenses, EA appears to promote a more balanced and controlled astrocytic response to inflammatory stimuli (Fig. 7). These findings provide evidence that EA modulates glial responses and contribute to a better understanding of its actions during neuroinflammation. However, further studies are needed to elucidate the underlying mechanisms and determine the translational relevance of these findings.

## Data Availability

No datasets were generated or analysed during the current study.

## Abbreviations

CAT: Catalase
CDNB: 1-Chloro-2,4-dinitrobenzene
CMF: Calcium-and magnesium-free buffer
CNS: Central nervous system
DCFH-DA: 2,7-dichlorodihydrofuorescein diacetate
DMEM: Dulbecco’s modified Eagle’s medium
DMSO: Dimethylsulfoxide
EA: Ellagic acid
EDTA: Ethylene diamine tetraacetic acid
FBS: Fetal bovine serum
GFAP: Glial fibrillary acidic protein
GSK3-β: Glycogen synthase kinase 3 beta
GSH: Glutathione
GST: Glutathione S-transferase
H_2_O_2_: Hydrogen peroxide
IL-1β: Interleukin 1 beta
IL-10: Interleukin 10
IL-6: Interleukin 6
LPS: Lipopolysaccharide
MAPK: Mitogen-activated protein kinases
MTT: 3(4,5-dimethylthiazole-2-yl)-2,5 diphenyl tetrazolium bromide
NF-κB: Nuclear factor kappa B
Nrf2: Nuclear factor erythroid 2-related factor 2
OD: Optical density
PAMP: Pathogen-associated molecular pattern
PBS: Phosphate buffered saline
qPCR: Quantitative real-time polymerase chain reaction
ROS: Reactive oxygen species
STAT: Signal transducers and activators of transcription
SH: Total sulfhydryl
SOD: Superoxide dismutase
SRB: Sulforhodamine B
TCA: Trichloroacetic acid
TNB: 2-nitro-5-thiobenzoic acid
TNF-α: Tumor necrosis factor-alpha
TLR4: Toll-like 4 receptor
